# Exploring Genetic Numeracy Skills in a Sample of U.S. University Students

**DOI:** 10.3389/fpubh.2017.00229

**Published:** 2017-08-29

**Authors:** Margo W. Bergman, Patricia Goodson, Heather Honoré Goltz

**Affiliations:** ^1^Milgard School of Business, University of Washington – Tacoma, Tacoma, WA, United States; ^2^Department of Health and Kinesiology, Texas A&M University, College Station, TX, United States; ^3^University of Houston—Downtown, Houston, TX, United States; ^4^Baylor College of Medicine, Houston, TX, United States

**Keywords:** patient communication, genetics, numeracy, young adults, genetic testing

## Abstract

Misconceptions concerning numerical genetic risk exist even within educated populations. To more fully characterize and understand the extent of these risk misunderstandings, which have large potential impact on clinical care, we analyzed the responses from 2,576 students enrolled at 2 Southwestern universities using the PGRID tool, a 138-item web-based survey comprising measures of understanding of genetics, genetic disease, and genetic risk. The primary purpose of this study was to characterize the intersection of risk perception and knowledge, termed genetic numeracy (GN). Additionally, we identify sociodemographic factors that might shape varying levels of GN skills within the study sample and explore the impact of GN on genetic testing intentions using both the Marascuilo procedure and logistic regression analysis. Despite having some college coursework or at least one college degree, most respondents lacked high-level aptitude in understanding genetic inheritance risk, especially with respect to recessive disorders. Prior education about genetics and biology, as well as exposure to biomedical models of genetics, was associated with higher GN levels; exposure to popular media models of genetics was inversely associated with higher GN levels. Differing GN levels affects genetic testing intentions. GN will become more relevant as genetic testing is increasingly incorporated into general clinical care.

## Introduction

In the years since completion of the Human Genome Project, the potential to improve health—spurred by innovations in information and technology—has not been fully realized (1). Yet the promises of personalized medicine to improve drug therapies (2), primary care (3), and patient empowerment (4) have captured both the lay public’s and health professionals’ imaginations. Geneticists, clinicians, and the public health workforce remain hopeful that integrating genetics into clinical practice and public health initiatives (as in personalized medicine and family health histories) will not only improve quality of care but will also alter health behaviors (3) and increase health equity (5).

As with any health-related innovation (6), genetics/genomics information and technologies embody several layers of complexity. For instance, critical challenges surround (a) the *intrinsic characteristics* of genetic-based information and technologies (4) and (b) *the lay public’s willingness and ability* to use genetic-based innovations. One characteristic of genetics-related information is the manner in which risk of developing a genetic-associated illness is conveyed to a lay client: genetic risk is almost always conveyed using statistical probabilities—despite established evidence that understanding and applying statistical probabilities are difficult tasks for large segments of the U.S. population (2–4).

The lay public’s willingness to adopt genetic innovations will depend on its ability to understand this probability-based information fully (7). Therefore, identifying whether population groups have adequate genetic literacy to understand genetic information and utilize this information to navigate accompanying services will be paramount for the upcoming era of personalized medicine/public health.

## Genetic Numeracy (GN)

Genetic numeracy is a subset of general health literacy (8). Scholars have characterized genetic literacy as “sufficient knowledge and appreciation of genetics principles to allow informed decision-making for personal well-being and effective participation in social decisions on genetic issues” (9). Genetic literacy, therefore, comprises both health literacy skills (those relating to the ability to read and write) and numeracy skills of a specific type (those necessary for estimating probabilities of outcomes, given specific parameters) (10).

Such numerical ability, combined with the conceptual knowledge required for understanding and interpreting genomic or genetic risk, we have labeled “genetic numeracy.” Although there is no universally accepted definition of GN, the research literature highlights the importance of having high-level fluency with numerical analysis, as well as basic understanding of DNA and the laws of inheritance (9, 11, 12), to master the knowledge base of genetics/genomics. This fluency is crucial for patient–provider discussions where providers convey the results of a genetic test, the risk of inheriting a genetic disorder, or the likelihood of developing a disease bearing a genetic origin (13–16). GN is equally crucial for non-clinical populations seeking to understand personal risk for developing illnesses based on family histories and/or current lifestyles (17).

## Background and Purpose

In a previous study (10), we examined how a convenience sample of undergraduate and graduate students from three Southwestern universities conceptualized genetics and genetic risk. Using a qualitative design and focus group methodology, we uncovered rather surprising findings regarding the explanatory models participants developed related to genetics and genetic risk. As part of our findings, we documented how “participants misinterpreted *numerical* genetic risk, visualizing each potential genetic risk as an independent event, rather than a dependent risk calculation.” (10).

We became intrigued by this divergence in understanding of risk based on the medical model of genetics/genomics and curious about which sociodemographic factors might be linked to such numeracy difficulties. Additionally, we began to question how prevalent these difficulties might be among this population. To explore this phenomenon more fully, we analyzed survey data from a larger non-probability sample of university students who took part in a broader study—the Perception of Genetic Risk in Sexual and Reproductive Decision Making by College Students (PGRID) (18). The choice to focus on university students was deliberate as, in theory, they have access to the most current genetics knowledge and up-to-date skills and tools for interpreting and integrating this knowledge. Moreover, compared to other age groups, this population is actively involved in both academic and/or professional training, perhaps as opinion leaders (19) in their communities, and in personal sexual and reproductive decision-making—rendering the topic both relevant and timely.

Few studies have explored the GN skills of specific populations, which sociodemographic factors might be associated with higher or lower numeracy, or how these different skills would impact genetic testing intentions. Previous quantitative studies in this area have explored genetic literacy and general numeracy separately in various contexts. For instance, Langford et al. (20) found that race, education, and socioeconomic status are predictive of knowledge of direct-to-consumer genetic testing. Kaimal and colleagues (21) showed that non-White race and lower educational attainment are associated with lower scores on numeracy and genetic literacy scales among pregnant women. Rolison et al. (22) found that poor numeracy is predictive of misunderstanding the difference between absolute and relative risk for prostate cancer. These differences in objective and subjective risks affect perceptions of provider communication quality, with patients possessing lower subjective numeracy also perceiving communication with providers as having low quality (23). When Vassy and colleagues (13) asked participants with lower numeracy skills about motivations for behavior changes based on hypothetical test results for type II diabetes, participants were more likely to state that lower genetic risk results would motivate more behavior changes. However, these behavioral intentions did not necessarily translate to actual behavior modification.

Given the sparse literature on the topic, therefore, our purpose in further analyzing data from the PGRID study was to characterize the GN skills of this university-aged sample and identify which sociodemographic factors might moderate these skills. Moreover, we tested whether the sample’s GN skills were associated with their (hypothetical) intention to obtain prenatal genetic testing and with their past experience with genetic testing. This manuscript reports these analyses and our findings.

## Materials and Methods

### Sample Recruiting

The current study reflects secondary analysis of the Perception of Genetic Risk in Sexual and Reproductive Decision Making by College Students (PGRID) dataset (18). The original data were collected using a 138-item web-based survey instrument. To recruit for the PGRID, we utilized a voluntary census of 68,125 students attending two Southwestern universities during the 2007–2008 academic year (students who had not affirmatively opted out of their school’s information directory). The original dataset contained responses from 2,576 students (response rate of 4%). For this study, we excluded respondents who did not answer items utilized in the GN scale (*n* = 1; see description of measure below), or only completed the demographic portion of the survey (*n* = 6). The final sample size was 2,569. The PGRID study was approved by Institutional Review Boards at each study site. As a secondary data analysis, this study was exempt from additional IRB approval.

### Measures

Given that we defined genetic numeracy as the numerical skills and conceptual knowledge required for understanding and interpreting genomic or genetic risk, assessing college students’ GN required a measure able to capture *both* the numerical *and* the knowledge dimensions of this definition. The measure we utilized was developed specifically for this study because existing numeracy measures had not been designed to capture these dimensions. Measures available at the time of data collection merely identified basic arithmetic skills (9, 14). We, therefore, created a GN measure comprising a composite score based on the answers to several genetic risk questions (requiring assessments of probability) in conjunction with scores on genetic knowledge questions.

To capture the numeric dimension in the original survey, the following scenario-based question was posed for different familial relationships: “If one of the following relatives were diagnosed with a genetic disorder, how would you rate your risk for developing the same disorder?” The relatives listed were: mother, father, sibling, aunt or uncle, nieces or nephews, grandparent, and the respondents’ (existing or hypothetical) children. Response options ranged from 0 to 100% risk, in 10% increments. Using standard Mendelian Punnett Square analysis (24) if only one parent was diagnosed with a recessive genetic disorder, the potential for inheriting that disease by a child should be 0%, excluding epigenetic or environmental interactions. For a dominant genetic disorder, the inherited potential risk is 50%. Similar determinations can be made for the various degrees of relatives.

During preliminary data analysis, we observed how respondents were inconsistent in their answers regarding probability of risk, based on familial relationships of the same degree of kinship. For example, one respondent indicated that while he/she perceived no risk from inheriting a genetic disease diagnosed in the mother, he/she indicated a 70% risk of inheriting a genetic disease from the father. To eliminate these inconsistencies, we computed in the GN composite score only answers to the question scenarios mentioning the mother (i.e., the risk of inheriting a genetic illness from the mother). We operated under the assumption that these responses would be more valid than responses to scenarios involving other degrees of kinship because research has shown both men and women have closer relationships with their mothers than their fathers (25), translating into greater communication between mothers and children about health risks and potentially more accurate knowledge of risk probabilities (26, 27).

To assess the knowledge dimension of GN, we employed a 5-item subscale of the PGRID survey instrument. The total knowledge score resulted from the sum of all correct answers (minimum = 0, maximum = 5). Therefore, in this study, respondents classified as having *High Genetic Numeracy* (HGN) have appropriate maternal risk perceptions and high knowledge scores (scores of 4 and 5 on the knowledge subscale). Participants classified as having *Low Genetic Numeracy* (LGN) have inappropriate maternal risk perceptions and low knowledge scores (0 or 1). The remainder of the sample was classified as having *Moderate Genetic Numeracy* (MGN). This group may have had good knowledge, but inappropriate maternal probability assessments, or had appropriate maternal probability assessments, but low knowledge scores.

We measured intention to undergo genetic testing with a single item—asking whether the respondent would pursue prenatal testing in the future. Past experience with genetic testing was evaluated by asking “Have you ever had genetic testing to determine if you carry specific gene(s) for a specific genetic disorder?” Female participants were also asked, “If you have ever been pregnant, did you undergo prenatal testing for genetic disorders?”

Health behavior theories propose that engaging in actions to improve health—such as undergoing genetic prenatal testing, particularly in cases of existing familial risk—can be influenced or moderated by perceptions of risk, perceived severity of the ensuing illness, beliefs about the benefits of the preventive action, and the actual (or perceived) barriers preventing a particular action (7). Both internal and external dynamics influence these factors, including education, information sources, and personal relationships (6, 7, 28). Based on propositions in the Diffusion of Innovations Theory (6), for this study, we hypothesized the following variables might moderate levels of GN: education—particularly, whether participants had taken classes focusing on biology, genetics, or genetics-related topics; whether participants had ever charted their family health history or family tree (genealogy); or whether they had ever undergone a genetic test. Additionally, we also measured which media sources participants had used to obtain genetics-related information: they were asked about the use of the Internet, newspapers, magazines, non-fiction books, TV, and radio. Although at the time, these data were collected social media was not as prevalent as it is currently, we also asked the sample whether they had ever talked about genetics with friends, relatives, personal physicians, or other health professionals. According to the Diffusion of Innovations Theory (6), communication channels and personal relationships play an important role in the spread of novel information among its potential adopters.

### Analyses

In this study, we examined the relationship between (a) GN and genetic testing intention, (b) GN and past experience with genetic testing, and (c) the relationship among GN, education, sources of genetics information (media and personal relationships), and genetic testing intention. We used Bonferroni corrected chi-square analysis, combined with the Marascuilo procedure (29), to analyze whether there are differences in either demographics or genetic testing intention among participants identified as having HGN, MGN, or LGN. The highly unequal distribution of the GN scores limited the ability to run logistic regression to predict differences in numeracy, along with sample size issues resulting from the large number of potential predictor and control variables. We also used logistic regression to identify factors associated with intention to engage in future genetic testing. STATA 13 was employed in all analyses (30).

## Results

### Sample Characteristics

Table 1 outlines the demographic characteristics of our sample (*N* = 2,569): one-fourth of the sample (25%) comprised graduate students; the remainder were either undergraduate students (68%) or students classified as “others” (7%; e.g., professional students). More than half of the participants were female (64%) and had an average age of 23.4 years (SD = 6.69). The largest ethnic group represented was White, with similar numbers of Hispanics/Latinos and other ethnicities (19 and 15%, respectively). Half the sample (52%) was unmarried. In terms of religiosity, half considered themselves Protestants (50%) and one fourth identified as Catholic (25%); 64% perceived themselves as “moderately” or “very” religious/spiritual, and 11% as “not at all” religious/spiritual. The practice of church attendance, however, yielded a slightly different pattern than that for perceived religiosity/spirituality, with 46% of the sample attending no church services on a weekly basis.

**Table 1:** Demographic characteristics of participants in the PGRID study classified as having High Genetic Numeracy (HGN), Moderate Genetic Numeracy (MGN), and Low Genetic Numeracy (LGN).

	LGN (*N* = 427)	MGN (*N* = 2060)	HGN (*N* = 82)	Overall sample (*N* = 2659)	χ^2^ (Bonferroni corrected, p* = 0.017)
	16.6%	80.22%	3.18%		
Gender					
Female	65%	64%	73%**	64%	0.219
Average age (σ)	23.2 (6.37)	23.4 (6.69)	22.6 (4.06)	23.4 (6.56)	
Ethnicity					0.002*
White	60%	67%	71%	66%	
Hispanic/Latino	25%**	18%**	9%**	19%	
Other	15%	14%	18%	15%	
Marital status					0.780
Single	54%	52%	37%	52%	
Religion					0.004*
Agnostic/atheist/none	15%	18%	26%	18%	
Catholicism	30%**	25%**	15%**	25%	
Protestant	49%	50%	46%	50%	
Others	6%	7%	13%**	7%	
Extent religious or spiritual					0.724
Not at all	9%	11%	16%	11%	
Slightly	27%	26%	23%	26%	
Moderately	42%	40%	39%	40%	
Very	22%	23%	22%	23%	
Weekly attendance at religious services					0.013*
0	42%	46%	56%**	46%	
1	43%	36%	32%**	36%	
2–4	13%	16%**	11%	16%	
5+	3%**	2%	1%	2%	
Educational status					0.017*
Undergraduate	68%	68%	68%	68%	
Graduate	25%	25%	30%**	25%	
Others	7%	7%	2%**	7%	
Enrolled in a Biological Sciences degree					
Yes	5%**	20%**	50%**	19%	0.000*
Taken a genetics course					
Yes	6%**	19%**	54%**	18%	0.000*
Taken a course with genetic info					
Yes	71%	86%**	94%**	84%	0.000*
Charted your family tree					
Yes	45%	50%**	38%**	49%	0.021
Charted your family health history					
Yes	36%	39%	46%**	39%	0.144
Taken a genetic test					
Yes	7%**	6%	4%**	6%	0.294
Read about genetics—Internet					0.001**
Never	36%	22%**	6%**	24%	
Not very often	37%**	34%	34%	34%	
Not often	15%	24%**	18%	23%	
Often	10%	16%	26%	15%	
Very often	2%	4%	16%	4%	
Read about genetics—newspapers					0.001***
Never	51%**	35%**	16%**	37%	
Not very often	33%	37%**	37%**	37%	
Not often	12%**	21%**	30%**	20%	
Often	3%	6%	11%**	6%	
Very often	1%	1%	5%	1%	
Read about genetics—magazines					0.001***
Never	43%**	30%**	15%**	32%	
Not very often	36%	36%	26%**	36%	
Not often	13%**	22%**	39%**	21%	
Often	6%	10%**	15%**	9%	
Very often	1%	2%	6%	2%	
Read about genetics—non-fiction book					0.001***
Never	55%**	38%**	22%**	41%	
Not very often	25%	31%**	27%**	30%	
Not often	14%	20%**	28%**	19%	
Often	4%**	8%**	17%**	7%	
Very often	2%	2%	6%**	2%	
Heard about genetics—TV					0.002*
Never	11%	8%**	2%**	8%	
Not very often	34%**	27%	26%**	28%	
Not often	27%**	33%	34%**	32%	
Often	22%**	26%	30%**	26%	
Very often	5%	5%	8%**	6%	
Heard about genetics—radio					0.018
Never	47%	40%	31%	41%	
Not very often	28%	34%	41%	34%	
Not often	17%	19%	20%	19%	
Often	6%	6%	4%	6%	
Very often	1%	1%	4%	1%	
Talked about genetics—friends					0.001***
Never	25%**	17%**	9%**	18%	
Not very often	34%	32%	25%**	32%	
Not often	27%	32%**	25%	30%	
Often	11%**	16%	15%	15%	
Very often	3%	4%	15%**	4%	
Talked about genetics—relatives					0.001***
Never	26%**	17%**	12%**	18%	
Not very often	28%	28%	21%**	28%	
Not Often	29%	32%**	27%	32%	
Often	13%**	19%**	29%**	18%	
Very Often	3%	4%	11%**	4%	
Talked about genetics—physicians					0.004*
Never	42%**	36%**	26%**	36%	
Not very often	24%**	30%**	32%**	29%	
Not often	20%	23%**	20%	22%	
Often	12%**	10%	20%**	10%	
Very often	2%	2%	4%**	2%	
Talked about genetics—other health professionals					0.216
Never	60%	58%	55%	58%	
Not very often	20%	22%	26%	22%	
Not often	12%	14%	15%	14%	
Often	6%	5%	1%	5%	
Very often	1%	1%	4%	1%	

*is significant at the .05 level.

**Indicates statistical significance via the Marascuilo Procedure.

***is significant at the .01 level.

Most participants had enrolled in a course containing genetics-related information (84%), and one in five participants was seeking a Biological Sciences degree or had taken a course in genetics (19 and 18%, respectively). Although almost half the sample (49%) had charted their family tree, fewer (39%) had developed a family health history. In terms of genetic testing, only a very small subset of the overall study sample (approximately 1 in 16 participants or 6%) had ever taken a genetic test.

Low percentages of the total sample (less than 10%) stated that they heard or read about genetics (in various media) “very often.” When asked whether participants talked about the topic of genetics with friends, relatives, physicians, or other health professionals, frequencies were high for the “never” and “not very often” categories. For example, 65% had “never,” or “not very often,” talked with physicians about the topic, and 80% had “never,” or “not very often,” talked with other health professionals about the topic. Nearly half (46%) of participants had “never” or “not very often,” approached the subject with their relatives; and half the sample (50%) “never” discussed it with their friends.

### GN Variability by Demographic Factors

Nearly one-fifth of the sample (16.6%) was classified as having LGN, and 3.18% as having HGN. The remaining 80.22% were classified as having MGN (see Table 1).

There were significantly more females in the HGN group (74%) than in the LGN (65%) or in the MGN (64%). GN scores did not differ by marital status or average age. The HGN group had a smaller number of respondents reporting they were of Hispanic/Latino origin (9%); conversely, the largest ethnic group in the LGN category was Hispanic/Latino (25%). Similar patterns emerged for the religious variables: 30% identified as Catholic in the LGN group, while 15% in the HGN group declared being Catholic. Significantly higher percentages of respondents in the HGN group identified as religious non-Christians (labeled “Other” in Table 1). Participants’ views of themselves as religious or spiritual (ranging from “not at all” to “very”) did not differ, but the number of church services they attended per week did. More than half (56%) of the HGN group attended no church services on a weekly basis, and 32% attended one weekly service. The LGN group had the highest number of participants attending five or more times per week (3%). Furthermore, levels of GN varied significantly by education status, with 30% of the HGN group being enrolled in a graduate degree, and 2% in a professional type degree. All levels of GN had similar numbers of participants with an undergraduate degree.

High Genetic Numeracy respondents were most likely, of all the groups, to either be enrolled in a Biological Sciences degree (50%), having taken a course in genetics (54%), or having taken a course with some genetic information (94%). LGN respondents showed a markedly different pattern, with 5% enrolled in a Biological Sciences degree, 6% having taken a genetics course, and 71% having taken a course with genetic information. Both LGN and HGN respondents were less likely to have charted a family tree when compared to the MGN group (45, 38, and 50%, respectively). HGN respondents, however, were more likely to have charted a family health history (46%).

Another pattern of differences among the three groups was their exposure to genetic information. The HGN group reported much lower levels of “never” reading or hearing about genetics in multiple contexts, with only 2% “never” hearing about genetics on TV, and 6% “never” reading about it on the Internet. The LGN group, by contrast, reported much higher proportions of “never” hearing/reading about genetics, whether from non-fiction books (55%), newspapers (51%), magazines (43%), the Internet (36%), or television (11%). The HGN group was also significantly more likely to have talked about genetics “very often” with friends (15%), relatives (11%), or physicians (4%), while the LGN group reports, again, large numbers “never” engaging in conversations about genetics with friends (25%), relatives (26%), or physicians (42%).

### GN, Past Experience, and Genetic Testing Intention

Using the Marascuilo procedure of multiple proportion comparisons (28), we analyzed whether the three groups LGN, MGN, and HGN differed in their previous genetic testing experience and future genetic testing intention (Table 2).

**Table 2:** Distribution of participants in the PGRID study classified as having High Genetic Numeracy (HGN), Moderate Genetic Numeracy (MGN), and Low Genetic Numeracy (LGN), according to past experience and future intention to undergo genetic testing.

Percentage	LGN	MGN	HGN	Overall sample
Have received—general	6.32	6.57	3.66^a^	6.27
Have received—prenatal	6.06	8.32	4.76^a^	6.46
Would receive—prenatal	57.08	59.20^a^	58.67^a^	57.49

*^a^Indicates statistical significance via the Marascuilo Procedure*.

Table 2 describes the distribution of respondents’ past experience and future intention to undergo genetic testing. The HGN was less likely to have undergone genetic testing (3.66%) or prenatal testing (4.76%), than the other two groups. They did, however, indicate significantly more willingness (58.67%) to receive prenatal testing in the future than the LGN group (57.08%).

To determine which sociodemographic factors might help explain the differences in genetic testing behaviors and intention, we ran a logistic regression with the various characteristics that we assessed in this study (see Table 3).

**Table 3:** Logistic regression of factors associated with future intention to undergo prenatal genetic testing among a sample of university-enrolled students in the U.S. (PGRID study).

Logistic regression		Number of obs = 2,476		
		LR χ^2^(60) = 235.09		
		Prob > χ^2^ = 0.0000		
Log likelihood = −1,592.43		Pseudo *R*^2^ = 0.0687		
Log likelihood (constant-only) = −1,709.97				
Correct% classification: 62.3				
Correct% classification (constant-only): 53.2				

**Future prenatal testing**				

**Variable**	**Adjusted odds ratio**	***p* > |*z*|**	**95% confidence interval**	

Age	1.004	0.605	0.988	1.021
Female*	0.801	0.019	0.666	0.964
Married*	1.328	0.001	1.115	1.580
Racial (others)				
Hispanic/Latino*	1.585	0.005	1.146	2.192
White	0.983	0.897	0.753	1.281
Educational status (undergrad)				
Graduate*	1.408	0.037	1.021	1.943
Professional	0.953	0.722	0.733	1.240
Others	0.752	0.166	0.502	1.126
Religion (none/atheist)				
Others	0.929	0.734	0.606	1.422
Catholic*	0.664	0.015	0.477	0.922
Protestant	0.808	0.169	0.596	1.095
Extent religious (not)				
Slightly	0.783	0.17	0.553	1.110
Moderately*	0.578	0.005	0.395	0.845
Very*	0.325	0.001	0.211	0.501
Weekly service attended (0)				
1	1.068	0.572	0.850	1.343
2–4	0.960	0.801	0.696	1.322
5+	0.288	0.513	0.385	1.611
Biological Sciences degree	1.275	0.061	0.989	1.645
Taken a genetics course	1.174	0.247	0.894	1.541
Taken a course with genetic information	1.009	0.944	0.795	1.280
Charted family tree*	0.824	0.032	0.691	0.983
Charted family health history	1.071	0.472	0.889	1.289
Read about genetics on the Internet (never)				
Not very often	1.020	0.882	0.781	1.333
Not often	0.914	0.581	0.664	1.258
Often	0.980	0.918	0.671	1.431
Very often	1.359	0.346	0.719	2.568
Read about genetics in a newspaper (never)				
Not very often	1.081	0.534	0.845	1.384
Not often	1.037	0.83	0.744	1.445
Often	1.361	0.214	0.837	2.212
Very often	3.276	0.121	0.730	14.696
Read about genetics in a magazine (never)				
Not very often	1.126	0.376	0.866	1.463
Not often	1.274	0.157	0.911	1.783
Often	1.189	0.437	0.769	1.837
Very often	1.151	0.76	0.466	2.844
Read about genetics in a non-fiction book (never)				
Not very often*	0.746	0.013	0.591	0.941
Not often	0.919	0.556	0.694	1.217
Often	0.956	0.824	0.644	1.419
Very often*	0.411	0.007	0.216	0.783
Talked about genetics with relatives (never)				
Not very often	1.124	0.444	0.833	1.516
Not often	1.161	0.366	0.840	1.605
Often	1.139	0.513	0.772	1.679
Very often	1.782	0.078	0.937	3.390
Talked about genetics with friends (never)				
Not very often	1.153	0.343	0.859	1.546
Not often	1.281	0.142	0.921	1.781
Often	1.247	0.281	0.834	1.864
Very often	1.641	0.132	0.861	3.127
Talked about genetics with physician (never)				
Not very often	1.031	0.792	0.823	1.290
Not often	1.233	0.116	0.950	1.600
Often	1.112	0.543	0.789	1.567
Very often	1.506	0.229	0.772	2.937
Heard about genetics on the TV (never)				
Not very often	1.098	0.603	0.772	1.560
Not often	1.027	0.888	0.711	1.482
Often	1.102	0.635	0.739	1.642
Very often	1.343	0.311	0.759	2.374
Heard about genetics on the radio (never)				
Not very often	0.889	0.283	0.718	1.101
Not often	0.915	0.533	0.691	1.210
Often	0.678	0.073	0.443	1.036
Very often	0.491	0.12	0.200	1.204
Numeracy (moderate)	0.889	0.283	0.718	1.101
Low	1.238	0.072	0.981	1.561
High	0.763	0.286	0.464	1.254

*is significant at the .05 level.

The model yielded three variables significantly associated with future prenatal testing: being married (AOR = 1.33, *p* < 0.05; single as reference); identifying as Hispanic/Latino (AOR = 1.58, *p* < 0.01; non-Hispanic/non-White as reference); and being a graduate student (AOR = 1.41, *p* < 0.05; undergraduate as reference). Seven variables were significantly associated with lower likelihood of receiving future prenatal testing: being female (AOR = 0.804, *p* < 0.05); identifying as Catholic (AOR = 0.663, *p* < 0.05; atheist/agnostic as reference); being moderately (AOR = 0.577, *p* < 0.05) or very (AOR = 0.324, *p* < 0.05) religious (“not religious” as reference); having charted a family tree (AOR = 0.824, *p* < 0.05); and having read about genetics in non-fiction books, either not very often (AOR = 0.746, *p* < 0.05) or very often (AOR = 0.411, *p* < 0.05; “never” as reference).

## Discussion

We assessed the GN levels of a sample of 2,569 university-enrolled students in the U.S., together with select sociodemographic characteristics of the sample and their associations with GN. We also examined the relationship between GN and past experience with genetic testing, as well as GN and future prenatal genetic testing intention. We focused our discussion on those variables that could be affected directly through programmatic interventions or indirectly by influencing the network of social relationships in which the individuals are embedded.

As anticipated, respondents who were Biological Sciences majors had taken a course in genetics (or a course with genetics information), or had charted a family tree had significantly higher HGN levels (Table 1). Unfortunately, our study design and analyses do not account for the “chicken-or-egg” phenomenon: we are unable to determine whether these factors lead to higher GN levels/skills or whether persons who have HGN seek out degrees, courses, sources of information, and personal interactions that reinforce or enhance their interests and abilities. Regardless of cause, our findings provide an opportunity to reflect on feasible practices that may either produce or reinforce high levels of genetic literacy, overall.

Recent research, for instance, highlights the influence of peers and various social norming agents (e.g., friends, relatives, physicians, and other health-care providers) on a person’s risk perceptions and on his/her general or genetics-related decision-making (31, 32). Peer influence, in particular, is more pronounced in the adolescent and young adult years (33) in part due to neurological changes in the risk–reward structures of the brain (26, 34). In our sample, the average age of respondents was 23 years. More than 94% of the sample was between 18 and 35 years of age, placing this sample firmly in the late adolescent/young adult category. This age range represents a prime target for assimilating innovative health information, especially given the sexual and reproductive decisions this group faces. Promoting easily accessible, accurate information and fostering environments in which debates or conversations about genetics-related topics can ensue may be cost-effective and feasible ways to enhance the health and genetic literacy of university-aged young adults.

Religion is another source of social norming that can shape genetic literacy (35), and our sample exhibited significant differences in GN across professed religious groups: among respondents who were identified as Catholic, only 15% had HGN (Table 1) and after controlling for various demographic factors, those identifying as Catholic were significantly *less likely* to accept prenatal genetic testing in the future. One possible explanation for the association between religion and GN is the perceived locus of control for health of these populations (36), especially for genetically linked illnesses. People who believe that disease is under control of a higher power are also less likely to take pro-active steps to improve their health (10, 37–39). It is possible, then, that respondents affiliated with more deterministic religious traditions will be more likely to view their genetic destiny as fixed, and therefore not be as interested in understanding something outside their control. Since numeracy levels differed by religion, in our sample, research exploring the dynamics of these religious factors and genetic literacy, overall, is sorely needed. Moreover, given our significant findings also suggesting a relationship among GN, genetic testing intention and ethnicity (Hispanics/Latinos had significantly more intention to test in the future), the interaction between ethnicity and religion may also be a factor worth exploring in further depth (40). Regardless of the precise dynamics at play, leaders within religious communities can play an important role in promoting genetic literacy by creating safe spaces for learning and debating the ethical nuances inherent in genetic technologies. Addressing potential dissonance between religious and scientific beliefs can enhance the health outcomes of entire population groups (41, 42).

Among other feasible leverage points within this system of interacting factors we examined, we observe that communicating with health providers can be another useful resource for enhancing genetic literacy and GN, although the exact methods used for doing so must be studied further. In turn, we argue, enhanced literacy/numeracy skills can lead to improved health outcomes (43), particularly when providers use patient-centered communication techniques (44). As documented in our study, 78 and 96% of participants in the HGN group declared that they did not talk about genetics with their physicians or with other health professionals, respectively (i.e., “never,” “not very often,” or “not often”).

This acknowledgment (that little exchange is taking part between clients and providers) suggests an important gap in communication about genetics knowledge and technologies which health care providers could help bridge. Communication tailored to a patient’s genetic literacy/numeracy level, in tandem with leveraging appropriate social norming agents and cues to action (45), may ensure patients learn accurate and potentially life-saving information regarding their risk for a genetic illness. To serve university-enrolled students, both primary care providers and student health centers must be trained specifically to interpret and coach patients’ charting of family health histories and provide appropriate referrals or testing for any potential conditions. While student health centers commonly offer sexual and reproductive healthcare, they may not offer genetic testing and counseling services. Thus, partnerships with larger medical centers and community agencies would provide a continuum of care for diagnosis, treatment, and health decision-making. And as we consider these strategies, it is useful to bear in mind that over half our sample (57.49%) expressed an intention to undergo prenatal genetic testing in the future, if provided the opportunity.

### Strengths and Limitations

The current study benefits from a large sample size and a variety of theory- and evidence-based variables that could moderate the GN skills of university-aged students. Our findings also contribute to the literature by allowing a more comprehensive view of the qualitative findings we obtained in our first study. If in that study we learned participants misinterpreted genetic risk, the current study teaches this misunderstanding, or the difficulties with the numerical dimension of genetic risk, are widespread: only 3.18% of participants exhibited evidence of strong GN skills in our sample (Table 1).

Yet despite this study’s contributions to the health literacy literature, its findings must be considered within the context of its limitations. One important limitation is the low response rate (4%). Although the response rate is low for the overall population size (*N* = 68,125), we designed the recruitment as an untargeted email invitation. With this strategy, some invitations are filtered as spam or are ignored as unwanted mail, even if the recipients may have wanted to complete survey (46).

Another limitation regards our ability to generalize our findings to a broader university-based or young adult population in the U.S. The study’s sample was predominately young, White, female, and single, mirroring the college-age population within the target geographic region; however, the sample was not randomly selected so findings cannot be generalized. Further work should consider utilizing probability samples and include more diverse groups of participants with varying ages, educational attainment levels, ethnicities, and marital or parenting status.

Finally, the measures we utilized in this study may have, inadvertently, generated unintended biases. By eliminating inconsistent responses and controlling for shared variance in our regression model, we attempted to minimize potential biases in the current analysis. However, further refinement and testing of the measures (particularly the GN composite scale) could help define optimal instruments for collecting more valid GN data from this and other population groups.

## Conclusion

The Secretary’s [of Health and Human Services] Advisory Committee on Genetics, Health, and Society (47) advises that ensuring the clinical utility of genetic and genomic testing is of the utmost priority as these technologies are further incorporated into health-care practice. Clinical utility depends on the ability of a test to change health behaviors or alter health outcomes (48), and changing health behaviors depends on changing health intentions (7, 28) alongside developing health literacy. Genetic test results, when not adequately understood, will lead either to no health behavior changes, or to inappropriate ones.

Genetic knowledge and GN will only become more important as we continue to further incorporate the research on the genome, the exome, the microbiome, and the interactions among them, ourselves, and our environment into daily medical practice. Much work remains to ensure that lay persons have the tools to interpret and act upon the information they receive about genetic inheritance, and to ensure that providers understand how, and why, patients interpret the information the way they do.

## Ethics Statement

This study is a secondary data analysis of data, and therefore exempt from human subjects approval.

## Author Contributions

MB contributed to the data analysis, data interpretation, initial paper draft, and paper revision. PG contributed to the initial data acquisition, data interpretation, and paper revision. HG contributed to the data acquisition, data interpretation, and paper revision.

## Conflict of Interest Statement

The authors declare that the research was conducted in the absence of any commercial or financial relationships that could be construed as a potential conflict of interest.
